# Why Do People with OCD and Health Anxiety Seek Reassurance Excessively? An Investigation of Differences and Similarities in Function

**DOI:** 10.1007/s10608-016-9826-5

**Published:** 2017-02-18

**Authors:** Brynjar Halldorsson, Paul M. Salkovskis

**Affiliations:** 1 Department of Psychology, University of Bath, Bath, UK; 2 School of Psychology and Clinical Language Sciences, University of Reading, Reading, UK

## Abstract

Excessive reassurance seeking (ERS) is commonly reported in patients who have OCD or health anxiety. Despite its prevalence and associated risk of ongoing difficulties, little is known about the function of ERS. It has been conceptualised as a type of compulsive checking behaviour, but could also be seen as being a supportive maneuver. This study offers a new approach towards defining ERS and support seeking (SS), and similarities between these two constructs in a sample of OCD and health anxious patients. A semi-structured interview was employed. Participants reflected on the nature and goals of their reassurance and support seeking—its impact on themselves and other people. Twenty interviews were conducted, transcribed and analysed in accordance to framework thematic analysis. Six overarching themes were identified in terms of ERS and five for SS. Results revealed limited diagnosis specificity of ERS. Strikingly, participants with health anxiety did not report seeking support.

## Introduction

Excessive reassurance seeking (ERS) is particularly prominent in people who suffer from obsessive compulsive disorder (OCD) and health anxiety (HA) (Abramowitz and Moore 2007; Kobori and Salkovskis 2013; Salkovskis and Warwick 1986; Salkovskis et al. 2003). ERS can be complex, persistent, extensive, debilitating and may dominate the interactions of those involved. From a cognitive behavioral perspective it has been hypothesized that ERS is a safety-seeking behaviour with the primary function of reducing perceived threat (Salkovskis 1996). Within this framework, reassurance seeking functions in a similar way to compulsive checking in OCD with the added potential of transferring ‘responsibility’ for the feared harm to another person (Rachman 2002; Salkovskis 1985, 1999). However, it could also be seen as being a supportive maneuver, and is often considered in this way by sufferers and their loved ones (Halldorsson et al. 2016). Despite the prevalence of ERS and the associated risk of ongoing difficulties, ERS remains under-researched, and, to our knowledge, only one study (Parrish and Radomsky 2010) has examined ERS using qualitative approaches. It is possible that empirical analysis into ERS has been hampered by a lack of adequate definitions of key concepts including ‘reassurance’ and ‘support’, as well as limited understanding of the difference between support, appropriate reassurance, and pathological reassurance seeking and giving of the type often clinically considered to be crucial to the maintenance of anxiety problems.

There is evidence to suggest that OCD is associated with a variety of interpersonal problems and, in turn, the interpersonal environment of individuals with OCD is an important factor for the progression and recovery of the disorder. For example, studies indicate that caregivers’ accommodating behaviours may impact on treatment outcomes (Amir et al. 2000; Garcia et al. 2010). However, it is important to keep in mind that caregivers suffer themselves as evidenced by, for example, elevated levels of distress, relationship difficulties and poor quality of life (Boeding et al. 2013; Torres et al. 2012).

In recent years, we have seen developments of ‘family assisted’ (e.g., Flessner et al. 2011), or more recently ‘partner assisted’ (e.g., Abramowitz et al. 2013), treatment interventions for OCD. To our knowledge, similar treatment developments have not taken place for health anxiety. However, with few exceptions (e.g., Abramowitz et al. 2013; Lewin et al. 2014; Renshaw et al. 2005), most family-based interventions focus on teaching family members to help with exposure based tasks as opposed to addressing directly interpersonal patterns or communications between family members.

With regards to the treatment of ERS specifically, the behavioral version of exposure and response prevention principles tends to inform clinical practice. Interventions usually take the form of instructing the patient to stop seeking reassurance while asking family members to withhold reassurance or ignore such requests (e.g. Abramowitz and Braddock 2008; Furer et al. 2001; Marks 2005; Rachman 2002; Taylor et al. 2005). However, recent studies examining ERS within the context of OCD have indicated that withholding reassurance can trigger strong negative behavioral and/or emotional reactions in OCD patients as well as increasing distress in caregivers (i.e. family members, partners and so on), therefore suggesting that ERS deserves a much better analysis and fine grained approach to intervention (Halldorsson et al. 2016; Kobori et al. 2012). This should not be surprising, as it is the *de facto* equivalent of turning water off in obsessional washers!

A notable exception is a recent pilot study where partners were encouraged to provide support in situations where the OCD patient felt overwhelmed with anxiety, i.e. “the partner provides support in ways the patient would like (but not using reassurance, rituals, or other accommodation behaviours)” (Abramowitz et al. 2013, p. 200).

With the interpersonal element of reassurance seeking in mind, Halldorsson et al. (2016) recently suggested that instead of focusing on ‘stopping reassurance’ it may be more effective to help patients to shift from seeking *reassurance* to seeking *support* —presented within a ‘theory A versus theory B’ framework (Salkovskis 1999). With this approach, patients are encouraged to substitute reassurance with a non-pathological interpersonal behaviour (i.e. support seeking) which acknowledges their distress without maintaining the perception of threat. To the best of our knowledge there are currently no published studies identifying the similarities and differences between reassurance seeking and support seeking and many questions remain unanswered about whether and/or how best to incorporate support seeking (as an alternative to reassurance seeking) into treatment. Furthermore, there appears to be no consensus about how best to define these concepts.

In the present study, we defined support seeking (and equally the provision of support) as:

*Interpersonal behaviour, verbal or non-verbal, that is intended to get (or give someone) encouragement, confidence or assistance to cope with feelings of distress*.

Thus, when a person seeks support the intention is to seek help to cope with distress and consequently this interaction is emotionally rather than threat focused, aimed as soothing acknowledged distress, including a sense that the person can accept or overcome their distress. This contrasts with the way in which the person experiencing severe and persistent anxiety does so because they believe that what is happening to them is more dangerous than it really is, and they have become ‘stuck’ in this belief (Salkovskis 1996). The patient is helped to consider and evaluate a less threatening explanation of what is happening (Salkovskis 1999; Salkovskis and Wahl 2003). By contrast, excessive reassurance seeking is defined here as:

*Verbal and*/*or non-verbal interaction with someone, who you perceive has access to potentially threat relieving information, with the intention of increasing your perceived sense of* *certainty* *of safety from harm*.

This definition can be modified to apply to specific disorders. The specific identification of ‘appraisals of responsibility’ as an additional motivational factor in OCD and HA is an example of this (Salkovskis and Forrester 2002). The responsibility factors are believed to overlap between these two disorders, but are not necessarily the same, i.e. there is some difference in specificity. In addition to dealing with the perception of threat, obsessional patients seek reassurance to disperse (or transfer) any/some responsibility of harm to others, whereas in HA the responsibility factors are less broad and are specifically focused on the person’s health and medical consultations where the individual intends to draw the attention of others to his or her physical state to allow for the detection of any abnormality (Salkovskis 1996). The aim with these new definitions is to provide a conceptual framework for studying the phenomenon of both concepts across disorders and assessing their psychological significance.

## Study Aims

To the authors knowledge there has been no systematic investigation into how ERS functions across different emotional disorders. Therefore, the aim of the present study is to provide insight into how OCD and HA patients understand ERS, specifically what motivates them to engage in these behaviours and how the behaviour impacts on themselves and other people. In addition, we aimed to explore how OCD and HA patients, understand and experience a different (potentially more helpful) interpersonal behaviour (i.e. support seeking), in order to examine the potential clinical utility of helping patients to shift from seeking reassurance to support.

This study is exploratory in nature and follows on from a previous study where caregivers of people suffering from OCD were interviewed about their experiences of reassurance seeking and giving (Halldorsson et al. 2016). The specific methodology employed in the present study was Thematic Framework Method, which offers a flexible and systematic approach to analyzing qualitative data and can be used to improve understanding of a phenomenon of interest, inform theory development and strengthen clinical practice (Gale et al. 2013; Ritchie et al. 2013).

Detailed hypotheses were not made, but instead general predictions were put forward derived from the cognitive behavioral theory of anxiety and the existing literature on ERS. They were:

The primary aims of reassurance seeking, in OCD and health anxiety, will be to prevent negative consequences such as threatened harm; as a secondary aim, will be the reduction of perceived responsibility.

The responsibility factors are believed to overlap between OCD and health anxiety with some difference in specificity.

The primary aims of support seeking will be to get someone’s help to cope with one’s distress.

## Method

### Participants

Thirty-five people completed the diagnostic screening process. Two participants were excluded based on a diagnosis of comorbid OCD and health anxiety. Two participants were considered to be in recovery from OCD and thus excluded. Three were excluded based on a diagnosis of GAD as their primary problem; three on the basis of suffering from major depression; and one on the basis of suffering from an eating disorder. Twenty-four were considered appropriate for the study, but four of them dropped out (3 OCD, 1 health anxiety). The final sample consisted of 20 participants (68.6 % acceptance rate). Participants were recruited from specialist anxiety clinics or self-help organizations in the UK. Participants were assessed by a qualified clinical psychologist using the Psychiatric Diagnostic Screening Questionnaire (PDSQ; Zimmerman and Sheeran 2003) and the Structured Clinical Interview for Diagnostic and Statistical Manual for DSM-IV Axis I Disorders (SCID; First et al. 1996). Participants were included in the study on the basis of meeting diagnostic criteria for OCD (and not comorbid HA; n = 10) or health anxiety (and not comorbid OCD; n = 10) and reporting seeking reassurance excessively within the context of their emotional problem. Other inclusion criteria were: (1) age between 18 and 70; (2) a good command of English. Exclusion criteria included: (1) The presence of severe psychopathology (e.g. psychosis); (2) risk issues (e.g. suicidality); (3) learning disabilities; and, (4) comorbid diagnoses of OCD and health anxiety. Demographic information is found in Table 1.

**Table 1:** Participants’ demographic and psychological characteristics

	Demographic characteristics							Psychological characteristics							
	ID	Gender	Age	Ethnic.	Employment	Relationship	Education	OCI	SHAI	PHQ9	GAD7	BAI	BDI	RAS	RIQ
OCD	Ppt. 1	F	46	White	Paid work	Cohabiting	Postgraduate	86	10	22	21	37	42	148	84
	Ppt. 2	F	31	White	Paid work	Single	Postgraduate	22	14	1	6	16	0	112	32
	Ppt. 3	F	32	White	Paid work	Single	Degree	38	15	2	9	20	12	131	4
	Ppt. 4	M	54	White	Paid work	Cohabiting	Degree	67	7	8	10	5	27	142	71
	Ppt. 5	F	26	White	Paid work	Married	Secondary	69	6	23	17	26	34	132	63
	Ppt. 6	F	26	White	Paid work	Single	Secondary	66	8	4	10	4	11	174	56
	Ppt. 7	F	26	White	Paid work	Single	Degree	49	4	4	15	21	8	127	43
	Ppt. 8	F	45	White	Paid work	Single	Secondary	105	22	15	14	12	19	173	70
	Ppt. 9	F	24	White	Paid work	Single	Degree	68	31	20	13	36	39	173	100
	Ppt. 10	F	27	White	Paid work	Single	Degree	82	15	9	8	8	33	161	54
Mean (SD)			33.70 (10.64)					65.2 (24.02)	13.20 (8.26)	10.80 (8.52)	12.30 (4.57)	18.50 (11.85)	22.50 (14.48)	147.30 (22.13)	57.82 (27.01)
HA	Ppt. 11	F	25	White	Paid work	Single	Degree	21	32	7	17	23	23	100	54
	Ppt. 12	F	33	White	Paid work	Cohabiting	Postgraduate	17	21	3	4	11	13	102	59
	Ppt. 13	F	40	White	Paid work	Married	Diploma	26	27	17	18	25	42	142	57
	Ppt. 14	F	29	White	Paid work	Married	Secondary	27	34	16	18	34	25	141	64
	Ppt. 15	F	41	White	Paid work	Cohabiting	Diploma	6	19	5	8	7	22	66	18
	Ppt. 16	M	29	White	Paid work	Cohabiting	Degree	19	20	14	13	21	35	115	40
	Ppt. 17	M	42	White	Paid work	Married	Diploma	32	35	16	8	16	10	105	^a^
	Ppt. 18	F	49	White	Paid work	Married	Secondary	16	40	23	20	44	45	141	61
	Ppt. 19	M	23	White	Paid work	Cohabiting	Diploma	91	41	22	21	58	38	127	63
	Ppt. 20	M	33	White	Paid work	Married	Secondary	19	41	11	15	32	40	120	56
Mean (SD)			34.4 (8.34)					27.70 (24.35)	31.00 (8.30)	13.40 (6.82)	14.20 (5.77)	27.10 (15.48)	29.30 (12.38)	115.90 (23.89)	52.50 (14.77)

*OCI* Obsessive Compulsive Inventory, *SHAI* Short Health Anxiety Inventory, *PHQ-9* Patient Health Questionnaire-9, *GAD-7* Generalized Anxiety Disorder-7, *BAI* Beck Anxiety Inventory, *BDI* Beck Depression Inventory, *RAS* Responsibility Attitude Scale, *RIQ* Responsibility Interpretation Questionnaire, *SD* standard deviation

^a^Missing data

### Materials

The semi-structured interview was split into two sections, one focusing on ERS and one focusing on SS. Participants were asked to, for example, describe what motivated and triggered their reassurance/support seeking, how they sought it, how it functioned, how they typically felt before/whilst receiving/after seeking reassurance/support, how helpful they found reassurance/support and what consequences (i.e. emotional and behavioural) it had when reassurance/support was withheld from them. The semi-structured interview schedule was developed with input from clinicians with expertise in treating emotional problems. After piloting the interview with clinical populations, the interview was shortened and slightly changed. The interview schedule can be obtained on request from the lead author.

In addition, participants completed eight self-report measures. *The Short Health Anxiety Inventory* (SHAI; Salkovskis et al. 2002) consists of 14 items, intended to measure health anxiety independently of physical health status. The instrument has demonstrated good reliability and validity (Abramowitz et al. 2007a, b; Salkovskis et al. 2002).

*The Obsessive Compulsive Inventory—Distress Scale* (OCI; Foa et al. 1998) is a well-established self-report measure within the OCD literature. It consists of 42 items which can be used for OCD diagnostic screening, severity testing and symptom profiling. The OCI has demonstrated high internal reliability and validity (Foa et al. 1998).

*The Patient Health Questionnaire-9* (PHQ-9; Kroenke et al. 2001) is a 9-item measure of clinically significant symptoms of depression. The internal reliability, factors structure, validity, and sensitivity to change have all been reported to be good (Cameron et al. 2008; Kroenke and Spitzer 2002; Kroenke et al. 2001).

*Generalised Anxiety Disorder-7* (GAD-7; Spitzer et al. 2006) consists of 7-items. Although it was designed primarily as a screening and severity measure of generalized anxiety disorder, it has also been found to be reasonably accurate in assessing panic, social anxiety and post-traumatic stress disorder (Kroenke et al. 2007). The scale has demonstrated good reliability and validity (Löwe et al. 2008; Spitzer et al. 2006).

*Beck Anxiety Inventory* (BAI; Beck et al. 1988) is a 21-item self-report inventory for measuring the severity of anxiety. It is typically considered the gold standard self-report measure of general anxiety symptoms.

*Beck Depression Inventory* (BDI; Beck and Steer 1987) is 21-item self-report inventory, intended to measure the severity of depression. The BDI is typically considered the gold standard self-report measure of depressive symptoms.

*Responsibility Attitude Scale* (RAS; Salkovskis et al. 2000) is a 26 item self-report measure that was administered to assess participant’s general assumptions, attitudes and beliefs about responsibility. The RAS effectively discriminates between people with OCD and both anxious and non-anxious controls. The scale has no clinical cut-off criteria. The RAS has demonstrated good reliability and validity (Salkovskis et al. 2000).

*Responsibility Interpretations Questionnaire—Belief* (RIQ-B; Salkovskis et al. 2000) is a 16-item self-report questionnaire, which was designed to assess respondent’s belief in their interpretations of intrusive thoughts. The scale has no clinical-cut off criteria. Test–retest reliability and internal consistency of the scale is good (Salkovskis et al. 2000).

All participant’s scores are reported in Table 1.

### Procedure

Once the participant’s eligibility was confirmed, participants were booked in for an interview (either in person or over the telephone). All interviews were administered by a qualified clinical psychologist (BH). Each session started with a summary of the study aims, that is, to discuss participant’s experiences and understanding of reassurance seeking and support seeking separately. In terms of the interview structure, participants were encouraged to elaborate on their answers, and give as much information as possible, avoiding a simple ‘yes’ or ‘no’ answer. The interviewer also prompted participants if he considered the answer required elaboration. Each interview lasted approximately 90 min and was recorded using a digital recording device and then transcribed verbatim (by independent transcribers). The interviews were coded and analysed according to the five stages of the thematic framework method (see below). Regular meetings between the research team (which consisted of the first author, two senior clinical psychologists and a research assistant) were held throughout the data analyses, allowing for further exploration of participants’ responses, discussion of findings, and agreement on recurring themes. Each participant received a £10 voucher. The protocol of the present study was approved by local Ethics Research Committee.

### Data Analytic Strategy

**Fig. 1:**
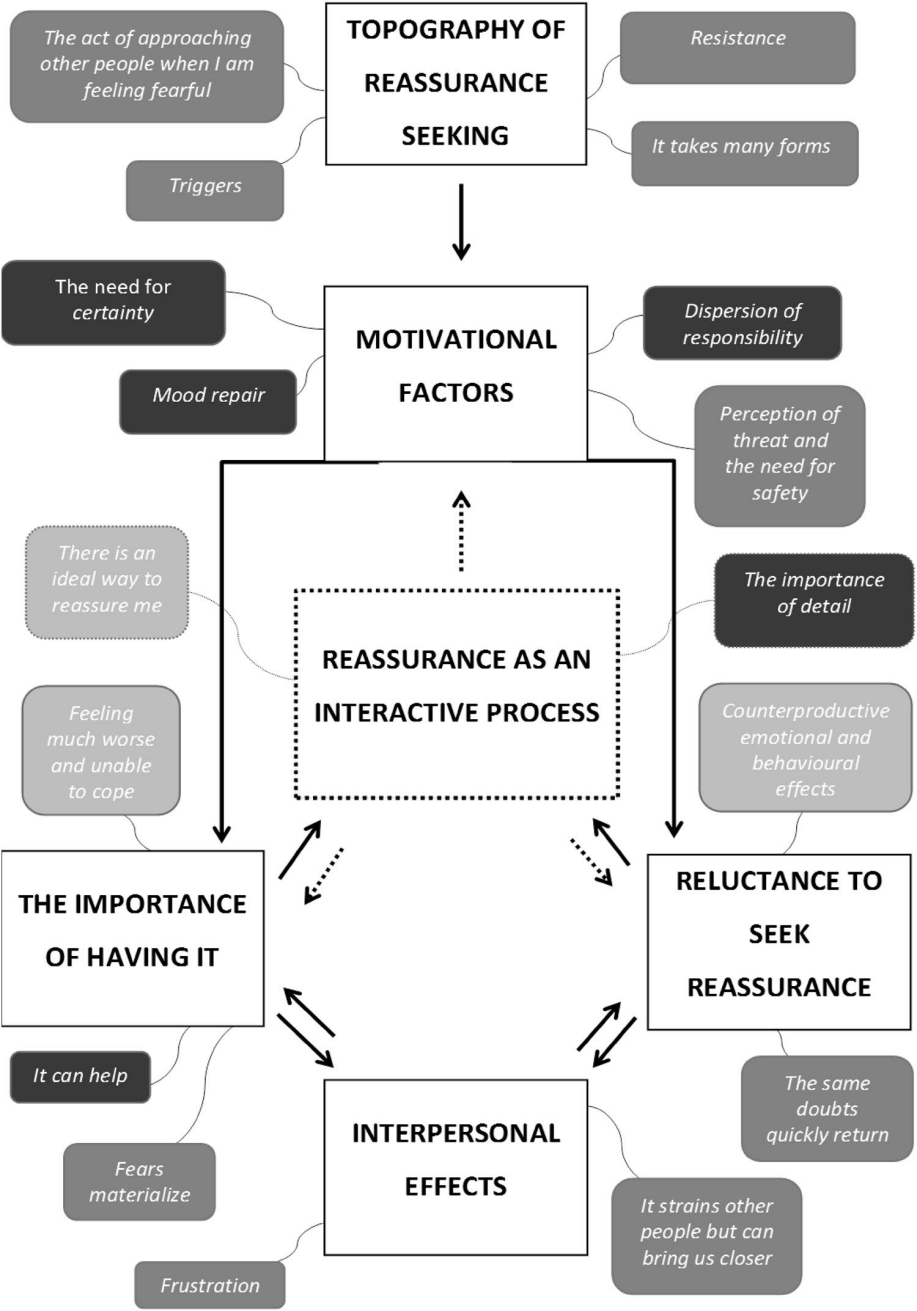
Thematic map of excessive reassurance seeking for OCD and health anxiety. *Note*: Each overarching theme is shown in *capital letters* and *bold*. Subthemes are presented in either,* dark grey*,* light grey* or* black color*.* Dark grey* (9 subthemes in total) reflects ‘no difference’; *Light grey* (3 subthemes in total) reflects ‘some difference’—meaning that both groups found that subtheme to be relevant but in a slightly different way; *black* (5 subthemes in total) reflects ‘difference’ between the groups—meaning that a considerable higher proportion of participants in one of the groups identified with that theme

**Fig. 2:**
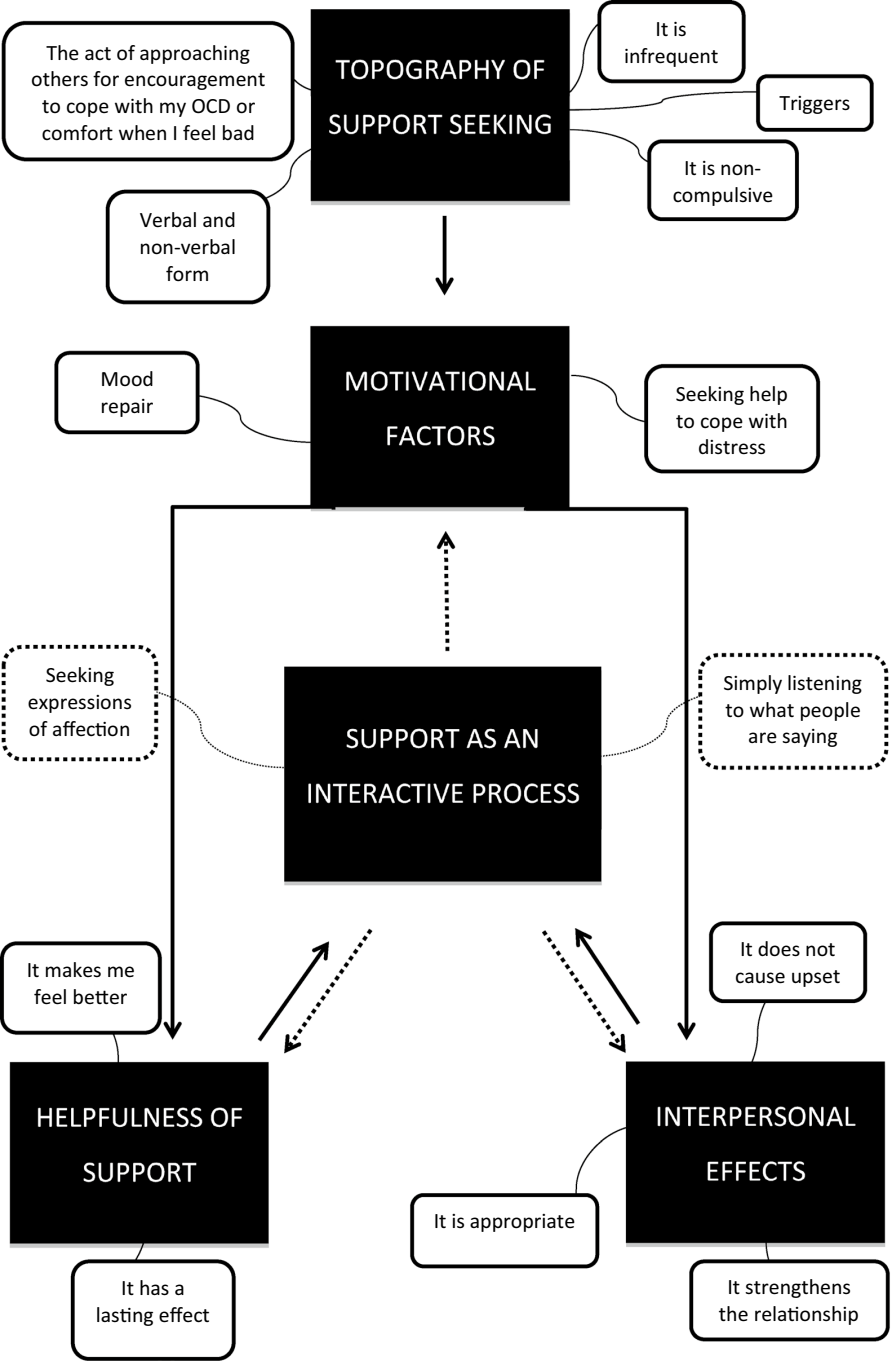
Thematic map of support seeking for OCD. *Note*: The 5 overarching themes that were identified are presented in *black rectangular shapes* and the 14 subthemes are presented in *white rounded rectangular* shapes

The current data set was coded and analysed using Thematic Framework Analysis (Gale et al. 2013; Pope et al. 2000; Ritchie et al. 2013). In accordance with the methodology applied the following five steps were taken: (1) *Familiarisation*: This step involved immersion in the data, listening to the tapes and thoroughly re-reading through transcripts in order to list key ideas and important and recurrent themes; (2) *Identification of a thematic framework*: Identifying all the key issues, concepts, and themes by which the data can be examined and referenced. This step was carried out by drawing on previous literature, theories and the specifics of the research questions. New themes were also identified from issues which the subjects raised themselves; (3) *Indexing*: This step involved applying the thematic framework systematically to all the interview transcripts; (4) *Data charting*: During the charting phase the data were lifted from their original context and rearranged and grouped according to the emerging themes; (5) *Mapping and interpretation*: Charts were reviewed, accounts of the two clinical groups were compared and contrasted, and connections made within and between codes, themes and cases/groups to explore relationships. This process was influenced by the original research objectives and by concepts generated inductively from the data. A thematic map was produced in order to summarise the findings visually (see Figs. 1, 2).

## Results

### Participants

Demographic status and psychological characteristics of both groups are presented in Table 1. On average, the OCD patients had been suffering from OCD for 19.9 years (SD 8.03) and the HA group had been suffering from health anxiety for 9.7 years (SD 3.20). The groups were similar in terms of age, ethnicity and employment status. Although there were more males in the HA group, Fisher’s exact test revealed that the difference was non-significant, *p* = 0.303. An independent samples t-test revealed no significant differences between the groups on self-reported depression (as measured by PHQ-9 *t*(18) = −0.753, *p* = 0.46; and BDI; *t*(18) = −1.129, *p* = 0.274) or anxiety (as measured by GAD-7; *t*(18) = −0.816, *p* = 0.42; and BAI; *t*(18) = −1.395, *p* = 0.180). The OCD group reported significantly higher scores on the OCI, *t*(18) = 3.467, *p* = 0.003, whilst the HA group reported significantly higher scores on the SHAI, *t*(18) = −4.681, *p* < 0.001.

### Overview of the Qualitative Analysis for Excessive Reassurance Seeking

In this section, we present the findings that relate to ERS. Both clinical groups were combined into one group for the analysis. Seventeen themes were identified which were aggregated into six overarching themes (see Fig. 1 for a thematic map).

#### Topography of Reassurance Seeking

This overarching theme includes four subthemes describing the phenomenological aspects of ERS.

##### Triggers

The OCD participants reported that the principal triggers of ERS were “doubts” (e.g. Ppt. 8; Ppt. 6; Ppt. 10; Ppt. 3), “anything to do with contamination” (Ppt. 5) or intrusive “thoughts” (Ppt. 2; Ppt. 4; Ppt. 7; Ppt. 9) typically with a harmful or violent theme: “I think when I feel- it’s like a sick anxiety…[and when] I have thoughts, I had one the other day, if I see a pregnant woman I think I’m going to punch her in the stomach” (Ppt. 1).

As expected, intrusive thoughts about health problems or catastrophic interpretations of physical sensations were most commonly identified as triggers for ERS among the HA participants: “I hold a lot of tension at the top, in my shoulders and in my chest. If I get any pain in that area, in my chest or- because obviously, that’s where my heart is then I will seek reassurance about that for sure” (Ppt. 15).

##### The Act of Approaching Other People for Help When I Am Feeling Fearful

All participants (OCD and HA) described ERS as an interpersonal behaviour involving approaching people (e.g. family members, doctors) or other sources (e.g. internet, books) they trusted for help whilst feeling fearful: “…just being able to get comfort from somewhere that you know it is just a thought and that nothing is going to happen as a result” (Ppt. 7, OCD); “It’s going back to people over and over again, for me it’s them telling me that everything’s ok and that I haven’t got anything seriously wrong with me” (Ppt. 18, HA).

##### Resistance

Eight OCD participants reported seeking reassurance daily, and all of them found it very hard (or impossible) to resist the urge of seeking reassurance: “Once it starts it’s very difficult to stop… It’s like once it starts it snowballs, it gets bigger and bigger and bigger. I really need to ask this question even though sometimes I think I know this is OCD” (Ppt. 1, OCD).

Similarly, eight HA participants said they sought reassurance daily and found it hard to resist: “… it just went out of control because I was constantly going to the doctors, constantly ringing my mum up or talking to my partner about what could be wrong with me it was just a constant, constant thing” (Ppt. 19, HA).

##### It Takes Many Forms

Nine OCD and nine HA participants reported that their ERS typically involved asking other people ‘direct questions’: “I will tell them the situation and ask if it’s something they would worry about or if it’s OCD” (Ppt. 5, OCD); “I say to him the most ‘do you think we ought to go to the doctor? Do you think I ought to ring the doctor?’ That is what I say to him every day nearly” (Ppt. 18, HA).

Participants in both groups also described seeking reassurance in subtle or hidden ways. However, this ‘form’ of ERS was less frequent compared to direct questions: “I’ll pretend that I’ve left something somewhere, or I’ll say if I’m not sure that I’ve turned the taps off in the bathroom, I’ll say ‘Oh look come and have a look at this, look, the tap’s doing something really weird’ and get them to come into the room, and then they’ll look at the tap and go ‘what are you talking about’ and I’ll go ‘Oh it’s stopped doing it now’ and then I’ll just walk away, and I know they’re the last one to have looked at the taps so it’s not my fault” (Ppt. 10, OCD).

All participants said they engaged in *self*-reassurance, but felt it was less effective in decreasing anxiety compared to receiving reassurance from other people: “I suppose I don’t always trust my own judgement whereas I trust the judgement of others” (Ppt. 15, HA).

#### Motivational Factors

This overarching theme consists of four subthemes which represent participants’ motivations for seeking reassurance.

##### Perception of Threat and the Need for Safety

Six participants in each group said they sought reassurance to prevent (or prepare for) a feared catastrophe: “So I know that nothing will happen to the house, burn down for example. To make sure it is safe” (Ppt. 9, OCD); “It’s just to take away that anxiety or to prove that you’re not going to die or that I don’t have a fatal terminal illness. Just to try and take away those fears” (Ppt. 17, HA).

##### Mood Repair

Five HA patients sought to change their emotional state when engaging in ERS: “Basically, just to feel better. Just to alleviate tension, anxiety” (Ppt. 20, HA). ‘Mood repair’ was mentioned by two OCD patients: “I would say to feel more comfortable, to feel calmer” (Ppt. 7, OCD).

##### Dispersion of Responsibility

Seven OCD patients associated ERS with the dispersion of responsibility: “[I seek reassurance] to avoid responsibility if something goes wrong” (Ppt. 10, OCD). The dispersion of responsibility was also mentioned by two HA patients: “So I seek reassurance that if there is something wrong with me someone will know what to do. So, that’s the feeling less responsible part, so I’m not the only one that’s responsible for what’s happened to me” (Ppt. 14, HA).

##### The Need for Certainty

Six OCD patients associated ERS with the need for certainty: “Because I need to hear it. Because I don’t trust myself. Because I might have it wrong and I kind of don’t trust my own opinion always yet. So, I suppose- Yeah I think that’s it. That I just don’t know, I still want to be told by someone else who knows better than me” (Ppt. 3, OCD). The need for certainty was unrelated to HA.

#### Reassurance as an Interactive Process

This overarching theme consists of two subthemes which describe what takes place whilst reassurance is being provided.

##### There is an Ideal Way to Reassure Me

Six HA patients and seven OCD patients talked about an ‘ideal way’ to reassure them. For the HA participants, ‘ideal reassurance’ typically involved experts such as doctors and/or medical tests: “I want scans done and blood tests and want all that lot done, because I want them to tell me that I’m actually really healthy, and that I’m fine” (Ppt. 19, HA). In contrast, none of the OCD patients associated ideal reassurance with experts and/or medical tests.

Similarities emerged between the groups in relation to how participants wanted reassurance to be delivered. Specifically, both groups felt that the ‘reassurer’ had to look confident and show that they had considered their question carefully, for example: “I don’t just want them to say it’s fine, you don’t need to do it, I want to feel they’ve listened and they’ve understood properly what I’ve said. And then they say it’s okay. I don’t want them to just say ‘Yeah it’s fine’ and they’re not actually listening. I want to know they have actually heard what I’ve said” (Ppt. 5, OCD).

##### The Importance of Detail

The groups differed on how attentive they were while reassurance was provided to them. Typically, the OCD patients said they listened very ‘carefully’ and paid close attention to various details (e.g. body language): “I listen very carefully… and usually I’m looking at them quite intently as well, just to try and get a genuine idea that they’re telling me the truth” (Ppt. 10, OCD). In contrast, the HA patients appeared to care less about *how* people answered them and even dismissing the reassurance almost immediately: “I listen to some extent, but not completely. I will probably, it’s almost like I’ll listen to myself more than anything, even if they’re talking, I’ll be like, if it’s not the answers that I want I’ll be like ‘That’s it, you’re not giving me the right answers, you’re not ever going to give me the right answers’” (Ppt. 19, HA).

#### The Importance of Having It

This overarching theme consists of three subthemes revealing a link between ERS and threat-relevant cognitions.

##### Fears Materialise

All participants said that when reassurance was either unavailable or withheld from them they typically became more fearful: “I would think that my thoughts would happen. That I would act on it and that I would do what my thought was telling me to do” (Ppt. 9. OCD); “…dying is the worst thing I could imagine actually” (Ppt. 11, HA).

##### Feeling Much Worse and Unable to Cope

Most HA patients also reported an increase in anxiety when reassurance was unavailable or withheld: “[I] go to pieces normally, and I think, I just give up” (Ppt. 20, HA). Interestingly, the OCD patients described a much broader emotional reaction, such as feelings of anger, frustration and abandonment: “Down-trodden, upset, anxious, maybe more anxious, fed up, frustrated, angry” (Ppt. 6, OCD); “I feel really, really angry. I feel completely abandoned” (Ppt. 10, OCD). When participants were asked to describe how they typically managed such circumstances (i.e. not getting reassurance), most participants said they continued to ask the person until they got what they wanted, or tried to find someone else to provide them reassurance.

##### It Can Help

All OCD patients said that reassurance typically made them feel better emotionally: “Overwhelmed with relief, lighter, like I’ve had weight taken off my shoulder, happier, yeah less stressed, less concerned. I actually feel a considerable, almost like a different person in a way” (Ppt. 6, OCD). In contrast, only half of the HA patients reported feeling any better after receiving reassurance.

#### Reluctance to Seek Reassurance

This overarching theme includes two subthemes reflecting why people with OCD and HA may be reluctant to seek reassurance.

##### Counterproductive Emotional and Behavioural Effects of Seeking Reassurance

Although reassurance typically appeared to provide participants some anxiety relief, they simultaneously recognized its counterproductive effects. Interestingly, the OCD patients tended to describe a much broader emotional/behavioural effect than the HA patients: “It’s… embarrassing. I’m embarrassed when I’m doing it, and I’m aware that I’m doing it and I feel embarrassed. So, I’m worrying about how I’m coming across” (Ppt. 1, OCD); “If I’m seeking reassurance and it doesn’t go the right way that I want it just makes me feel worse and the sensations in my body get worse and the panic gets worse as well” (Ppt. 19, HA).

##### The Same Doubts Quickly Return

Both groups consistently reported that the ‘positive’ effects of reassurance were typically short lived: “…it just wells back up in you. So kind of an immediate sense of relief and then it’s like getting grabbed in the guts again and it just comes back” (Ppt. 1, OCD); “I mean the initial ‘oh thank goodness’ you know, and then you know very often as soon as I’ve come out of the consulting room I think ‘oh I should’ve asked him about this’ or ‘I should’ve asked him about that, oh I’m going to have to make another appointment and I have to ask about that’ and of course that’s gone onto the next thing” (Ppt. 18, HA).

#### Interpersonal Effects

This overarching theme consists of two subthemes which reflect the negative and positive interpersonal effects of ERS.

##### It Strains Other People but Can Bring Us Closer

Overall, participants had mixed views about the short- and long-term *interpersonal* effects of ERS. Seven HA patients reported that ERS caused interpersonal problems in the short-term, for example, by putting “strain” on other people (Ppt. 15, Ppt. 16, Ppt. 17). Three HA participant did not associate ERS with any interpersonal problems (either short- or long-term) and four participants felt that their ERS had strengthened their interpersonal relationships.

Four OCD participants said that ERS had negative impact on their relationships with other people (both short- and long-term): “It has a really bad impact because it’s just, it’s frustrating for both of you, and I feel guilty about asking for it, and I feel weak for asking for it, and they feel frustrated for giving, and they know they’re not helping me really in the long run” (Ppt. 10, OCD). Six OCD participants associated ERS with *either* short- or long-term interpersonal problems. The same number of participants felt that their ERS had strengthened their relationships with other people.

##### Frustration

Five participants in each group reported that their ERS frustrated other people: “I suppose my partner can be a little, if it’s happened a lot, then he might get a little frustrated because what he’s saying I’m not necessarily listening to” (Ppt. 15, HA); “[X] gets frustrated with me. I think other people kind of tolerate it. They don’t like it but they tolerate it” (Ppt. 5, OCD).

### Overview of the Qualitative Analysis for Support Seeking

Interestingly, seven HA patients and one OCD patient were unable to identify examples where they had sought support within the context of their emotional problem—seeking reassurance was their ‘default’ interpersonal response. Consequently, the interview section that focused on SS had to be discontinued for these eight participants. On further discussion, the seven HA patients reported that they always sought reassurance when they felt health anxious. Five of them could not differentiate SS from ERS—to them reassurance was a form of support. The ‘absence of support seeking’ remained after the interviewer shared with the participants his definition of the concept and provided examples of support seeking, indicating that this finding does not result from a conceptual disagreement between the interviewer and the study participants.

What follows is a summary of the Thematic Framework Analysis that was carried out on the eight interviews that focused on support seeking within the context of OCD. The full analysis can be requested from they lead author. In sum, the OCD participants defined support seeking as an act of approaching others for encouragement to cope with their OCD or physical comfort when they felt bad. Typically, they reported seeking support infrequently and non-compulsively. Interestingly, the OCD patients associated this interpersonal behaviour with low mood and feelings of hopelessness as opposed to, for example, feelings of anxiety. With regards to motivational factors, the OCD patients said they sought support to change their emotional state (i.e. ‘repair their mood’) or get help to cope with their distress. In contrast to reassurance, support seeking does not appear to involve ‘careful’ listening and/or monitoring of the ‘reassurer’—it involves asking for/getting affection and empathy from someone you trust. Overall participants recognized support seeking as a helpful interpersonal behaviour—something they could use to fight their OCD—and people happily provided it. Additionally, participants felt that support seeking strengthened their relationships with other people. Findings are summarised in a thematic map (see Fig. 2).

## Discussion

This study sought to examine differences and similarities between excessive reassurance seeking and support seeking within the context of OCD and health anxiety. Reassurance seeking was present in both groups but strikingly, in comparison with the OCD patients, significantly fewer HA patients reported seeking support. With regards to ERS, both similarities and differences were noted between groups. Results indicate a shared topography of ERS across OCD and HA, specifically, all participants described ERS as a reaction to intrusive unwanted thoughts, doubts, images, anxious feelings or bodily sensations which were negatively interpreted. Furthermore, participants across both groups said that reassurance was difficult to resists, time consuming, and interfering. These findings are in line with what has previously been described in the literature (e.g. Abramowitz et al. 2003; Kobori and Salkovskis 2013; Parrish and Radomsky 2006, 2010; Salkovskis and Warwick 1986). Several motivational factors were identified. The results are consistent with the view that ERS is a reaction to the perception of threat—a behaviour which is intended to reduce perceived threat and/or seek safety, and in some cases, transfer feelings of responsibility onto others. In comparison to OCD, it was expected that the responsibility factors, in health anxiety, would be less broad and more specifically focused on the person’s health and medical consultations where the individual’s intention is to draw the attention of other people to his or her physical state to allow for the detection of any abnormality. However, this finding emerged in only two transcripts out of ten, possibly because this focus was not incorporated into the interview.

Other motivational factors were identified. In addition to reducing threat and dispersing/transferring feelings of responsibility, individuals suffering from OCD seem to engage in ERS to achieve a feeling of complete certainty. The ‘need for certainty’ is here understood as being an intolerance of uncertainty driven by the perception of threat; the person believes there is some concern that needs to be resolved—why would there otherwise be a need for certainty? This finding is in line with Wahl et al. (2008) who found that people with OCD made decisions to stop rituals on the basis of ‘elevated evidence requirements’. Kobori et al. (2012) reported that when OCD patients seek reassurance their focus is on transferring responsibility as well as the achievement of certainty that whatever they fear will not take place. This motivational factor seems less relevant for the HA patients who on the other hand were much more likely to engage in ERS to repair their mood.

In line with recent findings (e.g. Kobori et al. 2012; Parrish and Radomsky 2010) ERS was clearly found to be an interactive process. That is, when the OCD or HA patients are driven by these motivational factors, they try to make sure their criteria for ‘ideal reassurance’ is fulfilled by the ‘reassurer’. For the HA patients, ideal reassurance typically involved medical expertise. In contrast, the OCD patients were much less likely to want reassurance specifically from ‘experts’. The groups also differed on how carefully they listened to and how attentive they were whilst reassurance was provided. Whilst the OCD patients described paying close attention to various details (e.g. people’s facial expressions and how confident they looked) such process appeared not to take place amongst the HA patients.

The study suggests that withholding reassurance from OCD and HA patients may trigger negative emotional and behavioral responses, for example, increase feelings of distress, and this may occur in the absence of any tendency to seek support. This reaction is linked to the individual’s perception of threat and the need for safety. A relevant finding comes from Salkovskis and Kobori (2015) who showed that although the ‘positive’ effects of reassurance (anxiety reduction) diminish over the medium to longer term, OCD patients feel better after getting reassurance relative to not getting it.

It was of note how few participants seemed to be able to cope with circumstances where reassurance was not available or withheld. This reliance on reassurance becomes more interesting when we consider the effects of receiving reassurance when requested. Interestingly, half of the health anxiety group participants reported not feeling any better after getting reassurance. In contrast, all the OCD patients said they typically felt better when they were provided with reassurance. There are other disadvantages to reassurance seeking, which are more centered on interpersonal problems. Specifically, both groups reported that their requests for reassurance frustrated other people in the short-term but it tended to vary between patients whether they related ERS with negative long-term interpersonal problems. Interestingly some participants in both groups felt that their ERS had strengthened their interpersonal relationships.

The most striking finding from this study is how few of the health anxious participants reported seeking support within the context of their anxiety problem. Why would health anxious patients not seek support? The answer to this question is probably not straightforward, but egosyntonicity may be a key issue. Health anxious people consider their illness fears to be rational, thus they do not try to ignore or suppress their health fears because it makes sense to them that they have (or will have) health problems. In addition to that, most other people can to some extent relate to health fears and the need for reassurance under such circumstances (Salkovskis and Warwick 1986). Consequently, reassurance seeking could become a default interpersonal response to the perception of health threats and the associated distress, as opposed to other responses, including support seeking. In contrast to health anxiety, the egodystonic nature of obsessions calls for a different response to the perception of threat. Individuals with OCD recognize, by definition, that their beliefs are definitely or probably not true (or that they may or may not be true). This may suggest that theory B (a non-threatening alternative explanation) is already embedded in how the individual understands his obsessional problem. Thus, seeking support may perhaps automatically become an option. Furthermore, caregivers of OCD patients may find the process of reassurance particularly frustrating and do not relate to the sufferer’s fears like in health anxiety. Therefore, they may not understand the reasons (or find them bizarre) for why reassurance is sought from them and thus are reluctant to give it (Kobori and Salkovskis 2013). For these reasons, it is possible that the OCD patients were more likely than the HA patients to report seeking support within the context of their emotional problem. However, it seems likely that the full answer is much more complicated.

This study has several limitations. As with other qualitative research it can be criticised for its focus on narratives provided by a relatively small sample of patients. Consequently, an important limitation relates to the generalisability of the findings. A future study would benefit from a larger sample in addition to recruiting participants from more varied ethnical backgrounds (the current sample was limited to a white population). Furthermore, although the qualitative approach may be helpful in improving the understanding of a phenomenon of interest, there is a risk of researcher’s bias when the data is interpreted. Although steps were taken to address this issue (e.g. frequent expert supervision) it did not involve another researcher who independently coded the entire data set to allow for a more thorough comparison.
